# An epidemiological perspective on severe asthma

**DOI:** 10.1186/2045-7022-5-S2-O4

**Published:** 2015-03-23

**Authors:** Roxana Mincheva, Bo Lundback, Jan Lotvall

**Affiliations:** 1 Krefting Research Centre, University of Gothenburg, Gothenburg, Sweden

## Background

Extensive description of severe asthma phenotypes has gained much attention in the latest years with the hope of achieving better management of this potentially problematic group of asthmatics. However, all previous studies have focused on patient cohorts, and not random population samples.

## Aim

To dissect the concept of severe asthma, and to determine whether clustered complaints or addressing individual complains, will give more coherent understanding of severity.

## Methods

From a large population based study in West Sweden, a subgroup of 744 people with active asthma was characterised with extensive clinical examinations and detailed questionnaires. In this cohort we defined different parameters of severity as follows –1) low lung function as FEV1% predicted below 70%, 2) multi-symptom asthma described day-time symptoms, 3) 2 or more night-time awakenings per month, 4) use of rescue medications 3 or more times per week, 5) any respiratory emergency visits and 6) use of oral steroids regularly or on exacerbations in the last year. We outlined particular subgroups of asthmatics who presented with different extend of overlap of these characteristics and looked into possible risk factors for the separate groups.

## Results

The group of asthmatics who presented with at least one of severity features comprised of 374 people (50.3% of the active asthma group). The distribution for the individual complaints was day-time symptoms in 196 (36.3%), low lung function in 85 (11.4%), rescue medications in 130 (17.5%), night-time symptoms in 111 (14.9%), emergency visits in 78 (10.5%) and use of oral steroids in 66 (8.9%). When at least two complaints in a different combination were examined, the groups comprised of 2.1 -7.6% of the whole asthma population. The extent of the overlap between more than three complaints revealed tightly agglomerated groups representing 0.5 – 7.9% of all asthmatics. The risk factors that were outlined for the different groups were current smoking and having BMI > 30 with ORs ranging from 1.7 – 4.1 and 1.6 – 2.6, respectively.

## Conclusion

Further dissecting into the different severe asthma phenotypes in a random population could identify particular influencers for severity, and may more exactly stratify distinct groups that require extensive management.

